# Molecular Aggregation of Marketed Recombinant FVIII Products: Biochemical Evidence and Functional Effects

**DOI:** 10.1055/s-0039-1688413

**Published:** 2019-05-08

**Authors:** Raimondo De Cristofaro, Monica Sacco, Stefano Lancellotti, Federico Berruti, Isabella Garagiola, Carla Valsecchi, Maria Basso, Enrico Di Stasio, Flora Peyvandi

**Affiliations:** 1 Haemorrhagic and Thrombotic Diseases Service, Area of Hematology, Fondazione Policlinico Universitario “A. Gemelli,” IRCCS, Rome, Italy; 2 Institute di Internal Medicine and Geriatrics, Catholic University School of Medicine, Rome, Italy; 3 Fondazione IRCCS Ca' Granda Ospedale Maggiore Policlinico, Angelo Bianchi Bonomi Hemophilia and Thrombosis Center and Luigi Villa Foundation, Milan, Italy; 4 Institute of Biochemistry and Clinical Biochemistry, Università degli Studi di Milano, Milan, Italy; 5 Department of Pathophysiology and Transplantation, Università degli Studi di Milano, Milan, Italy

**Keywords:** recombinant FVIII, molecular aggregation, Hemophilia A, dynamic light scattering, FVIII inhibitors

## Abstract

**Background** Recombinant (rec-) coagulation factor VIII concentrates available for hemophilia A (HA) treatment differ in cell line production and structure, which could affect their pharmacodynamics and immunogenicity. Clinical trials showed that previously untreated patients with severe HA present higher rates of inhibitor development if treated with rec-FVIII products and that differences do exist as to inhibitor's formation among different rec-FVIII products. This finding could arise from several causes, such as absence of von Willebrand factor, different glycosylation profiles, or processes of molecular aggregation of the recombinant FVIII molecules.

**Objectives/Methods** In this study, using size exclusion high-performance liquid chromatography (SE-HPLC), dynamic light scattering (DLS) spectroscopy, and functional biochemical assays, we investigated the purity grade, FX activating ability, and aggregation status of three recombinant marketed products (Advate [Baxalta], Refacto AF [Pfizer], and Kogenate [Bayer]).

**Results** The overall analysis of the results obtained with SE-HPLC and DLS spectroscopy showed that the three recombinant FVIII concentrates contain low but significant amounts of molecular aggregates. This phenomenon was less evident for the Advate product. Molecular aggregation negatively affects the in vitro pharmacodynamics of the concentrates with higher aggregates' content.

**Conclusions** This study shows that the three pharmaceutical formulations of recombinant FVIII contain variable amounts of molecular aggregates after their reconstitution at therapeutic concentrations. This phenomenon negatively affects the in vitro potency of the products with higher aggregates' content and might be invoked as a contributing cause of their increased risk to induce the formation of FVIII inhibitors.

## Introduction

Patients with hemophilia A are currently treated with FVIII concentrates prepared with both recombinant technology and fractionation/purification from plasma of healthy donors. Recombinant FVIII products are produced by different cell lines, which synthesize FVIII molecules with the same primary sequence of the human factor (except the B-deleted and B-truncated molecules). However, these recombinant molecules inevitably go through different posttranslational modifications, such as glycosylation and tyrosine sulfation processes. 1 Furthermore, the process of expression and purification of recombinant FVIII products may potentially cause the accumulation of misfolded and aggregated proteins. These aspects may be responsible for perturbation of the efficiency and safety (regarding the inhibitor formation) of the recombinant products, as suggested in official documents by regulatory agencies. 2 The purification process of recombinant FVIII products includes a solvent/detergent virus inactivation step in addition to the use of ion exchange chromatography, and monoclonal antibody immunoaffinity chromatography to remove contaminating substances. 3 4 Chemical stabilizers such as amino acids, sugars, and nonionic surfactants are added for the maintenance of the structural/functional integrity of recombinant FVIII products. 3 It has been questioned whether the production of FVIII in nonhuman cells and the manufacturing processes could induce structural changes in the FVIII molecules and whether this might be the cause of different properties of products in terms of immunogenicity. Previous findings from randomized clinical trials (RCTs) and national hemophilia registries provided evidence that recombinant FVIII products are associated with high risk of inhibitor formation and that the recombinant second-generation FVIII products were associated with an even higher risk of inhibitor formation than the third-generation recombinant products. 5 6 7

In this study, we investigated some biochemical properties and, using size exclusion high-performance liquid chromatography (SE-HPLC) and dynamic light scattering (DLS) spectroscopy, the aggregation status of three recombinant concentrates belonging to the second (Kogenate) and third-generation products (Advate and Refacto AF). We addressed the issue of whether the molecular aggregation status of these products in solution after their reconstitution could significantly differ among products, affect their activity, and be invoked as a potential cause of inhibitor formation in hemophilia A patients.

## Materials and Methods

### FVIII Products

Three recombinant products (Advate [Baxalta/Shire], Refacto AF [Pfizer], and Kogenate [Bayer]) were studied. Three different lots of each product were used. In some experiments, Recombinate [Baxalta], the first-generation product of Advate and thus containing albumin, was also studied. FVIII preparations were reconstituted in distilled water for injection and passed through the particle filters (5 µm) contained in the pharmaceutical kit. The samples were immediately used for the experiments described below.

### UV Spectra of Recombinant FVIII Preparations

Ultraviolet (UV) absorbance scans of reconstituted Advate, Kogenate, and Refacto and genuine polysorbate 80 (TWEEN 80, purchased from Merck), histidine (25 mM, purchased from Sigma-Aldrich), and PEG 3350 (U.S. Pharmacopeia [USP] Reference Standard, Sigma-Aldrich) were performed over a 220 to 340 nm wavelength range in a 1-cm-pathlength quartz cell lodged in a thermostated cell holder of a Cary 60 spectrophotometer (Agilent Technologies Italia S.p.A. Milano, Italy). All the products were reconstituted with sterile water for injection by the use of the kits supplied by the manufacturers for therapeutic delivery and used at a final concentration of nominal 3,000 IU/mL (≅ 0.6 mg/mL, considering an activity of ≅ 5,000 IU/mg for the FVIII reference). The approximate concentrations of the above products were measured based on the extinction coefficient at 280 nm using *ε*_(01%)_ = 1.3 for the full-length FVIII products Advate and Kogenate, 8 and *ε*_(01%)_ = 1.55 for the B-deleted FVIII Refacto AF. 9

### SE-HPLC of the Recombinant FVIII Concentrates

FVIII samples of reconstituted Advate, Kogenate, and Refacto at a final nominal concentration of 400 IU/mL (≅ 80µg/mL) were analyzed by SE-HPLC using a TSK gel Super SW3000 column coupled to a two-pump apparatus (Jasco Easton, Maryland, United States), equipped with a spectrophotometric device (model 2075), and a spectrofluorometric detector (FP-2020, Jasco). The spectrophotometric detection of the eluted peaks was accomplished at 280 nm, whereas the fluorescence of the proteins was monitored by using λ _ex_ = 280 nm and λ _em_ = 340 nm. The elution buffer was a solution containing 20 mM phosphate buffer, 0.15 M NaCl, and pH = 7.40. The flow rate was 0.4 mL/min in all cases, and the injection volume was 75 µL. The same FVIII preparation was also filtered through a low-binding protein Millex-GP 220 nm filter (Merck) and analyzed again by SEC-HPLC, as detailed above.

### Dynamic Light Scattering Measurements of FVIII Concentrates

The outlines of the theory related to DLS techniques are described in biophysics textbook. 10 We shall only briefly describe the aim of our experimental strategy. In these experiments we used nominal concentrations of the rec-FVIII products usually employed for therapeutic infusions, corresponding to 100 to 250 IU/mL (≈20–50 µg/mL; ≈50–125 nM, considering a m.w. of full-length FVIII ≈275 kDa). At these concentrations, verified spectrophotometrically at 280 nm, the different rec-FVIII could not be detected in DLS spectroscopy. This bias was further complicated by the presence of a relatively high concentration of the excipients and stabilizers that actively contributed to the DLS signal. However, if molecular aggregates of rec-FVIII were present in solution, getting very high molecular mass, these objects should be detected in DLS spectra, even at a low concentration, as the signal's intensity is proportional to *d* 6 (where, from Rayleigh approximation, *d* is the hydrodynamic radius of a spherical particle). Hence, DLS intensity spectra of the genuine major excipients/stabilizers present in the various preparations (polysorbate 80, histidine, PEG 3350, mannitol, and threalose) were separately measured and compared with the DLS spectra obtained with the individual rec-FVIII products that contained the same stabilizers at similar concentrations. From this comparison, it was possible to detect and attribute components in solution that could be likely assigned to aggregated particles of the rec-FVIII molecules, as microscopic particulate was eliminated by the filters contained in the pharmaceutical kits. The DLS measurements were accomplished with a Zetasizer Nano S instrument (Malvern Instruments Ltd, Worcestershire, United Kingdom), equipped with a 4 mW He-Ne laser (633 nm) at 25°C at an angle of 173 degrees for determining the hydrodynamic diameter of molecular species in FVIII preparations. 11 12 13 The following recombinant FVIIIs powdered preparations were used: Refacto AF (Pfizer), Advate (Baxalta), Recombinate (Baxalta), and Kogenate. The powders were all reconstituted in sterile water and gently mixed until completely dissolved. Serial dilutions in sterile water occurred immediately after resuspension to analyze each preparation in the range of 100 to 250 IU/mL (≅ 20–50 µg/mL ≅ 50–125 nM) in a 45 μL quartz cuvette of 10 mm light path (Hellma Analytics, Germany). Reported data were acquired at the therapeutic concentration of 100 to 250 IU/mL and confirmed at different concentrations (data not shown). The intensity autocorrelation function (see the legend to Fig. 4 ) was analyzed using version 5.0 of the Zetasizer Nano Software (CONTIN algorithm). 11

### Cleavage of FVIII by Thrombin: Western Blot Method

The three rec-FVIII preparations (final concentration: 100 IU/mL, ≅ 20 µg/mL ≅ 70 nM) were reacted with 2.5 nM human thrombin (final concentration), purified as previously detailed, 14 in 5 mL of a buffer containing 50 mM Tris (pH 8.0), 150 mM NaCl, 2.5 mM CaCl _2_ , and 0.1% 2-mercaptoethanol at 25°C, according to a previous report. 11 Proteolysis by thrombin was monitored by Western blotting of the A2 domain (aa 375–719) of FVIII, recognized by the mouse monoclonal antibody (MoAb) sc-73597 (Santa Cruz Biotechnology, Heidelberg, Germany), as detailed below. Reactions were performed at 25°C for 30 minutes and, in some cases, up to 180 minutes. Proteins were electrophoretically transferred (45 minutes, 100 V) to a polyvinylidene difluoride membrane in Tris-buffered saline (TBS) 10X. Blocking was performed overnight at 4°C in 10% (w/v) nonfat dried milk in TBS with 0.1% Tween 20 to reduce the likelihood of false positives. Incubation was performed with antibodies against the full-length-FVIII (FL-FVIII) molecule. The anti-FL-FVIII monoclonal antibody sc-59508 (Santa Cruz Biotechnology, Heidelberg, Germany) recognizes a structural epitope of the entire intact FVIII molecule (native factor VIII A2 domain of human origin). A different mouse monoclonal antibody, sc-73597 (Santa Cruz Biotechnology, Heidelberg, Germany) raised against factor VIII of human origin, with epitope mapping to the A2 domain residues 497–510 and 584–593 was used in experiments investigating FVIII activation by thrombin. The membranes were then incubated with the secondary antibody, HRP-linked antimouse IgG NA931 (GE Healthcare, New Jersey, United States).

### Activation of FVIII by Thrombin: Functional Assays

For thrombin activation, recombinant FVIII samples were reconstituted at a concentration of 100 IU/dL (≅ 20 µg/mL ≅ 70 nM) into 50 mM Tris-HCl, pH 7.5, 150 mM NaCl, 2.5 mM CaCl2, 5% glycerol and incubated at 25°C with 20 nM human α-thrombin. 14 At timed intervals (0.5–20 minute), samples were taken from the mixture, and the activation was terminated by hirudin (20 nM, purchased from Sigma-Aldrich, units/mL) and diluted appropriately for FVIIIa activity determinations. 10 µL of these samples were diluted in 990 µL of human FVIII-deficient plasma from Werfen (Milano, Italy) and assayed for FVIII activity by the two-stage chromogenic assay, as described above. The presence of very low thrombin and hirudin concentration in the final solution did not affect the activation of FX.

Since the kinetic curves for the activation of the various FVIII products showed bell-shaped profiles, we analyzed the experimental data using a simplified kinetic scheme:



corresponding to:



In the kinetic scheme 1a, B represents the concentration of activated FVIIIa associated with the formation of FXa at any time of the reaction, whereas C reflected the inactivation of FVIIIa at any time. 15 The analysis of the generation of FVIIIa curves in these studies was based on the following phenomenological approach. In the activation phase, no attempt was made to use a model with any mechanistic connotation; only an empirical description was accomplished. Although it is known that FVIII activation by thrombin could be described at least minimally by Michaelis–Menten kinetics, analytical integration of the Michaelis–Menten function is impossible. Thus, although calculated by this simplified scheme, the overall time course of generation of FVIIIa at any time, [FVIIIa] _*t*_ , is given by the following exponential equation:



where [FVIIIa] _max_ is the final zymogen FVIII activated and *k*_1_ and *k*_2_ are pseudo-first order rate constants.

### FVIII Activity Assays

FVIII activity was determined by different methods:

- *One-stage clotting assay* : The assays were performed on an automated coagulation instrument (Top 700, Instrumentation Laboratory, Milano, Italy). The standard FVIII calibration curve was prepared with the Normal Plasma Calibrator from Werfen. This procedure was used for all products but Refacto AF, whose concentration was measured with the two-stage chromogenic assay alone (see below). Before determination, the samples were diluted at least 2,000-fold to concentrations that fall within the linear range of the FVIII calibration curve. Each sample was analyzed in duplicate, and the results were averaged.
- *Two-stage chromogenic FVIII activity* : FVIII chromogenic assay detection kits were purchased from Chromogenix (Werfen Group, Milano, Italy). The activity of generated FXa was measured at 405 nm, using the S2765 chromogenic substrate and a Top 700 instrument.

### FVIII Antigen Assay

FVIII antigen levels were measured using the commercial Kit ASSERACHROM FVIII:Ag (STAGO, Asnieres-sur-Seine, France). Control plasma (100 VIII:Ag IU/dL by comparing with the International Standard) was used as the reference. The assay sensitivity was 0.1 IU/dL FVIII:Ag.

### Assay to Measure Total Albumin Concentration

Albumin content of Kogenate was determined by an automated immunoassay (Hitachi 704/Roche Albumin Reagent).

## Results

### UV Spectra of Recombinant Products

**Fig. 1:**
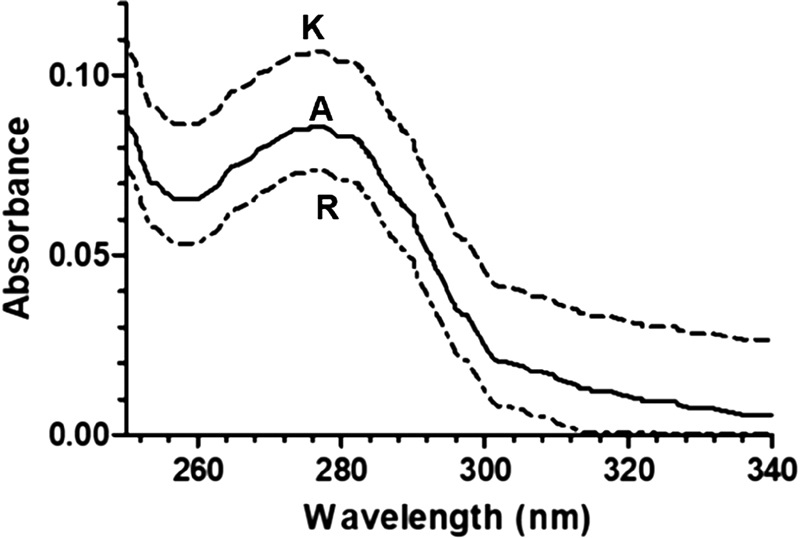
UV absorbance spectra of recombinant FVIII products: Advate, Kogenate, and Refacto were reconstituted in sterile water for the therapeutic injection, and absorbance spectra were obtained at 0.1-nm intervals in a thermostatic (25°C) 1-cm-pathlength cuvette lodged in a cell holder of an Agilent Cary 60 spectrophotometer. Each product was reconstituted with sterile water for injection at a nominal concentration of 3,000 IU/mL. A: Advate; R: Refacto; K: Kogenate.

The main characteristics of the recombinant FVIII formulations used in this study are listed in Table 1 . All the recombinant products showed typical protein absorbance peaks at ≈280 ± 1 nm attributable to tryptophan and tyrosine residues and a modest shoulder at 288 nm arising from tryptophan's contribution alone, as shown in Fig. 1 . The spectra were not significantly affected by Tween 20 and polysorbate 80, present as stabilizers in the Advate, Kogenate, and Refacto AF preparations. Genuine Tween 20, polysorbate 80, PEG 3350, and threalose at the final concentration of 0.1 mg/mL, 0.2 mg/mL, 3 mg/mL, and 2 gr/dL, respectively, showed an absorbance at 280 nm ranging from 0.003 to 0.008 AU, respectively. Based on the absorbance at 280 nm, the FVIII concentration of Advate and Refacto was calculated to be 0.66 ± 0.07 and 0.497 ± 0.06 mg/mL, respectively. It must be reminded that the protein content of Kogenate at 280 nm concerning FVIII per se was only apparent (0.84 ± 0.2 mg/mL) due to the presence of albumin in the solution. The above-reported FVIII concentrations were similar (except for Kogenate) to those measured by ELISA: Advate and Refacto AF showed a concentration of 0.58 ± 0.09 and 0.39 ± 0.04 mg/mL, respectively, while the Kogenate concentration was equal to 0.55 ± 0.06 mg/mL.

**Table 1:** Main characteristics of the three recombinant Factor VIII products used in the study

Characteristic	Advate	Kogenate	Refacto AF
Name of marketing authorization holder	Baxalta/Shire	Bayer	Pfizer Limited
Generation	Third	Second	Third
Engineered cell line	CHO (Chinese hamster ovary)	Baby hamster kidney (BHK)	CHO (Chinese hamster ovary)
Co-expression of VWF in engineered cell line	+	−	−
Primary sequence	Full length	Full length	B-domain deleted
Molecular weight	≈275 kDa	≈275 kDa	≈170 kDa
Virucidal treatment	Solvent/detergent	Solvent/detergent	Solvent/detergent, nanofiltration
Purification process	Ion exchange chromatography, monoclonal antibody immunoaffinity chromatography	Ion exchange chromatography, monoclonal antibody immunoaffinity chromatography	Immunoaffinity chromatography
Stabilizers and excipients	• Tris (hydroxymethyl) aminomethane • Calcium chloride • Mannitol • NaCl • α,α-Threalose • Histidine • Glutathion (reduced) • Polysorbate 80	• Sucrose • Calcium chloride • NaCl • Histidine • Glycine • Polysorbate 80 • Imidazole, tri-n-butyl, phosphate, and copper	• Sucrose • Calcium chloride dihydrate • L-Histidine • Polysorbate 80 • Sodium chloride

Abbreviations: VWF, von Willebrand factor.

### SE-HPLC of the Recombinant FVIII Concentrates

**Fig. 2:**
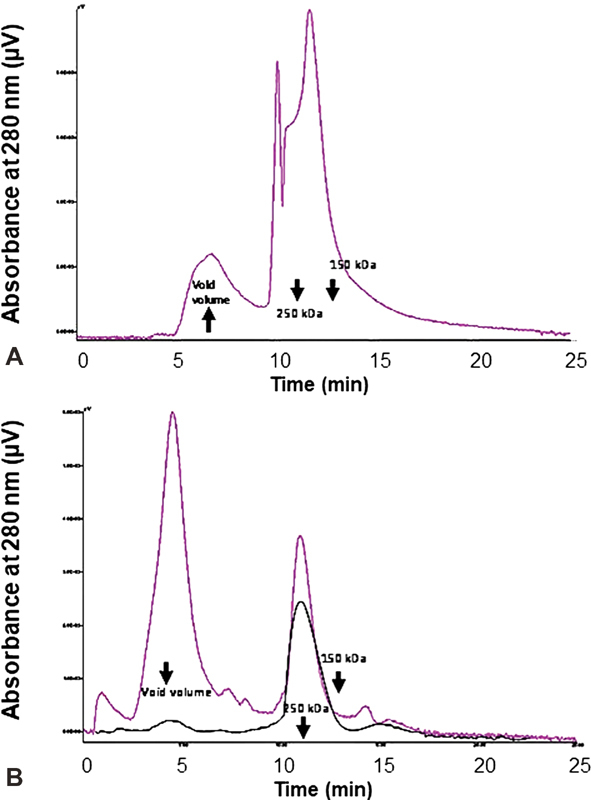
( **A** ) SE-HPLC of Advate (400 IU/mL) eluted with 20 mM phosphate buffer, 0.15 m NaCl, pH 7.40; ( **B** ) SE-HPLC of Kogenate (400 IU/mL) before ( *purple line* ) and after ( *black line* ) passage through a Millex-GP 220 nm filter (Merck). Note the almost complete elimination of the aggregated proteins after filtering the solution of Kogenate. The same result was obtained with Advate and Refacto AF. The molecular weight markers (250 and 150 kDa) and the void volume (protein material with m.w. > 1,000 kDa) are indicated by the *arrows* . SE-HPLC, size exclusion high-performance liquid chromatography.

The chromatographic analysis of the recombinant FVIII concentrates by SE-HPLC showed in all products the presence of significant amounts of very high molecular weight proteins, which were eluted in the void volume, which contained any protein with m.w. >500 kDa ( Fig. 2A ). This finding was particularly evident in Kogenate. Notably, in all cases, a filtering procedure through Millex-GP 220 nm filters eliminates almost completely the aggregated proteins ( Fig. 2B ). We tried unsuccessfully to quantify with precision the relative amount and molecular weight of the aggregated/unfolded material in the recombinant FVIII formulations by SE-HPLC. Unfortunately, this aggregated/unfolded proteinaceous material was eluted in the void volume (containing proteins with m.w. >500 kDa) so that the cumulative peak did not allow a precise and reliable calculation of the molecular weight of the proteins ( Fig. 2A ).

### Activation of FVIII Products by Thrombin: Electrophoretic Studies

**Fig. 3:**
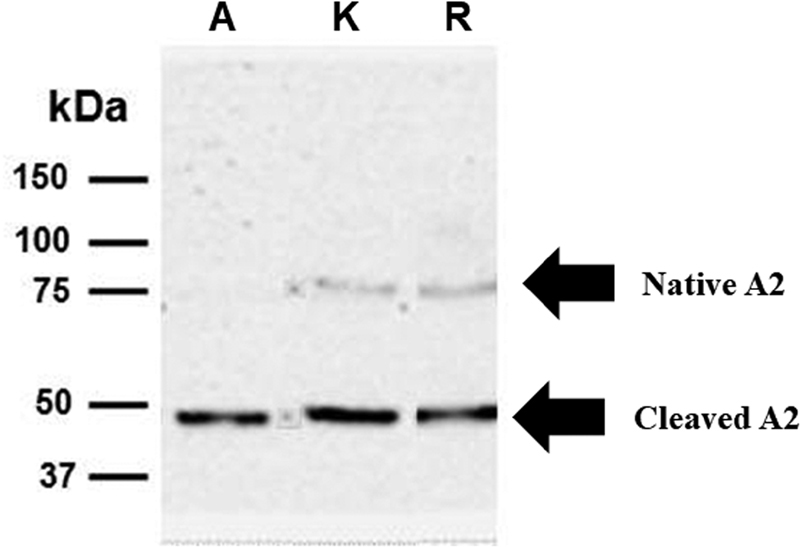
Thrombin-catalyzed activation of recombinant FVIII contained in Advate (A), Kogenate (K), and Refacto AF (R). The experimental conditions are reported in the Methods section. The detection of the A2 domain was accomplished by the MoAb sc-73597. Thrombin was used at a concentration of 2.5 nM, and the hydrolysis products were analyzed after 180-minute reaction.

Proteolysis of FVIII by thrombin was successfully monitored by Western blotting using a MoAb mapping of an epitope contained in the A2 domain of FVIII. Under the experimental conditions used in these experiments, thrombin hydrolyzed the entire amount of the A2 domain of Advate generating a fragment of ∼50 kDa approximately 15 minutes after thrombin addition, as shown in Fig. 3 . A similar pattern was observed for Recombinate (not shown) which derives from the same cell type (CHO) and the same gene construct, although it is a first-generation product and contains significant amounts of human albumin, which renders less clear the final gel of the Western blot. The other recombinant products (Kogenate and Refacto) were efficiently proteolyzed by thrombin, although after 15 minutes a little amount of the native A2 domain was still present ( Fig. 3 ). The A2 band was still present even after 180 minutes since the start of the thrombin addition (data not shown).

### Dynamic Light Scattering

**Fig. 4:**
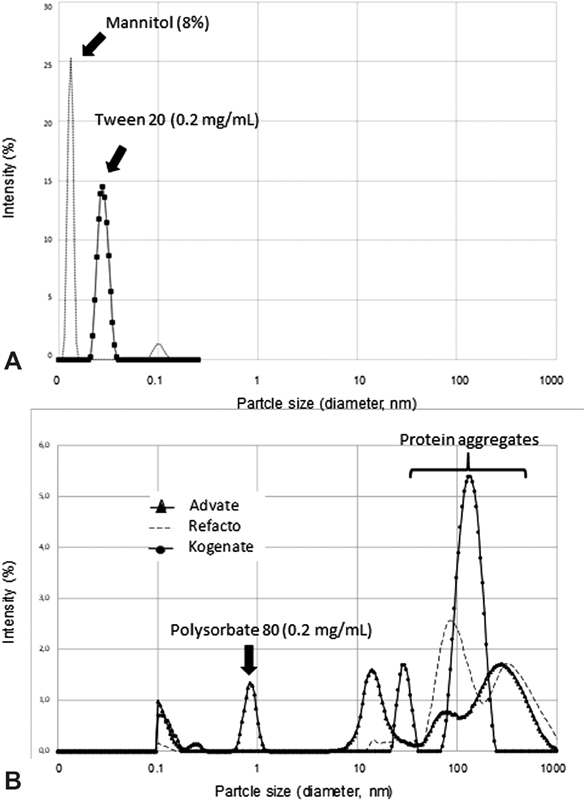
DLS measurements on rec-FVIII preparations and solution of genuine excipients and stabilizers present in the therapeutic preparations. All samples were equilibrated for 300 seconds. at 25°C just before the measurements start. The figure shows a size distribution of the particles by intensity measurements. ( **A** ) The distributions of the intensity of light scattered as a function of the diameter of Tween 20 (genuine solution: 0.2 mg/mL) and mannitol (genuine solution: 8%). Note the absence of particles with a higher hydrodynamic diameter. ( **B** ) Overlapping of the lines concerning the distribution of the intensity as a function of the diameter of the particles present in the Advate, Refacto AF, and Kogenate solutions. The large aggregates (indicated by *a brace parenthesis* ) were removed by filtration on a membrane with a pore diameter of 220 nm ( *continuous line* ), while particles with *d* < 100 nm were not altered by filtration. DLS, dynamic light scattering.

To better characterize the properties of the recombinant FVIII products (Advate, Recombinate, Kogenate, and Refacto AF), we also performed DLS measurements. It must be reminded that Recombinate contains human albumin in the final preparation at a concentration of 0.4 mg/mL at a nominal FVIII concentration = 100 IU/mL. The concentration range of FVIII preparations used in the DLS experiments (100–250 IU/mL) was chosen to reflect realistic concentrations of FVIII solution obtained upon reconstitution of the powders commercially available for the pharmaceutical preparations. The profiles of the autocorrelation function, *G* ( *t* ), for each FVIII product (data not shown) used in DLS experiments suggested the presence of polydisperse systems. 7 This result obliged to apply the multiple decay analysis (CONTIN) to determine the hydrodynamic radius of the molecules in solution. From the hydrodynamic radius and shape factors, it is possible to establish the size of the particle. 7 Size information is extracted from the measured correlogram by exponential fitting, where the particle diffusion coefficient *D* is proportional to the lifetime of the autocorrelation curve according to the following expression: *G* ( *t* ) = *B* + Σ *A* ·exp ^(−2· *q* ^2· *D* · *t* )^ (where *B* is the baseline, *A* is the amplitude, and *q* is the angle and wavelength dependent scattering vector). The hydrodynamic radius is then calculated from the diffusion coefficient using the Stokes–Einstein equation. 10 A theoretical hydrodynamic radius of 7 and 15 nm is expected for a globular and a linear molecule model, respectively, considering a molecular weight of 275 kDa for monomeric full-length FVIII molecules. In each preparation, at least two groups of components were identified: the first, with a hydrodynamic radius spanning from 7 to 30 nm, could be tentatively attributed to FVIII in a mono- and oligomeric form, while the second, spanning from ∼50 to 1,000 nm likely reflected the existence of unstable aggregates, partially unfolded or high molecular weight forms of FVIII. The second group of components peaking at >100 nm present in each preparation, and particularly in Kogenate, entirely disappeared after passage through filters with 220 nm cutoff. The range of the nominal concentration of rec-FVIII products used in these experiments would correspond to approximately 20 to 50 µg/mL (assuming a FVIII-specific activity of ∼5,000 IU/mg). Thus, at these nominal concentrations and assuming a molecular mass of ~275 kDa for full-length FVIII, the monomeric recombinant products could provide a backward DLS intensity signal very low or virtually absent in a Zetasizer Nano S instrument. The fact that we obtained significant DLS signals pertaining to objects with dimensions much higher than expected for FVIII molecules (up to 600 nm of hydrodynamic radius) represents a proof that protein aggregates are present in the pharmaceutical solutions. It is essential to keep in mind that there is a very strong dependence of the intensity of light scattered, with respect to particle diameter. These two values have a sixth-power relationship, which means, for example, that a 100-nm particle scatters one million times as much light as a 10-nm particle. Moreover, the signal pertaining to objects with very high hydrodynamic diameter could not derive from the excipient/stabilizers present in the pharmaceutical preparations. Genuine excipients such as polysorbate 80, Tween 20, or mannitol at the same concentrations present in the preparations (polysorbate 80: 50–200 µg/mL; mannitol 8% [w:v]) did not produce in fact any significant signal in the DLS apparatus pertaining to particles with diameter >1 nm ( Fig. 4A ). This result resembled what was observed in SE-HPLC experiments where, after a passage of rec-FVIII products through filters with a cutoff of 220 nm, the very high m.w. proteins were eliminated.

### Two-Stage Chromogenic Assay of Recombinant FVIII Products

**Fig. 5:**
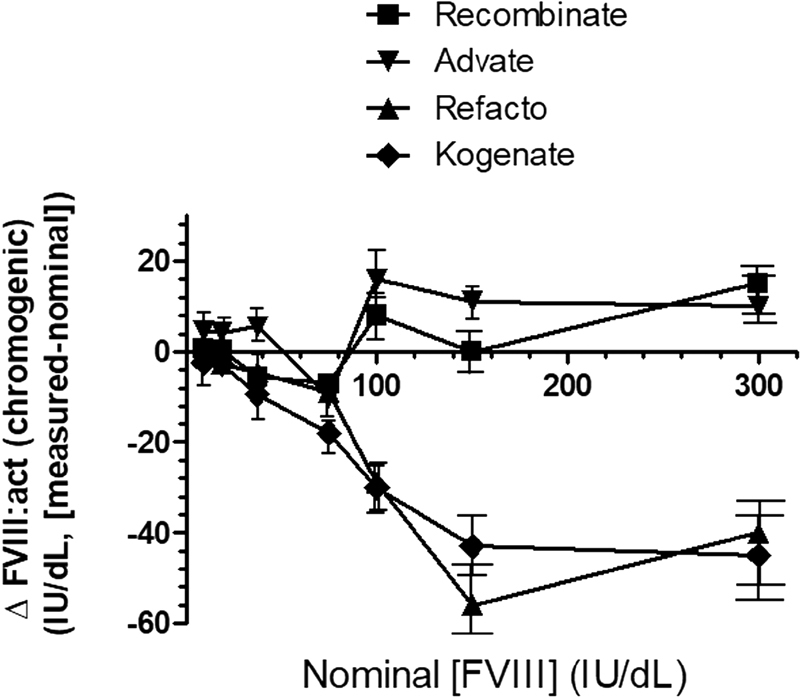
Difference between the nominal FVIII concentration of the products used in this study (x-axis) and the corresponding concentration (y-axis) measured with a chromogenic assay. The values are the mean of three different lots of each product. The *vertical bars* represent the standard errors from three different measurements from three different lots of the product. The intraassay standard error was 8 ± 3%.

The possible formation of high-grade FVIII aggregates that could affect the activity of FVIII was further explored by a functional assay, where the FX activating ability of each FVIII product, measured by a two-stage chromogenic assay, was investigated and compared with the nominal FVIII activity certified by the manufacturers. To do that, four recombinant FVIII concentrates were studied over a concentration range spanning from 9.4 to 300 IU/dL. The 13th British Standard for Blood Coagulation Factor VIII Concentrate was used as the reference (code labeled 10/188). In all cases, for samples with nominal FVIII concentrations >150 IU/dL, appropriated dilutions were performed to measure FVIII levels falling in the linear portion of the calibration curve. The final concentration was calculated considering the dilution factor. As shown in Fig. 5 , Advate and Recombinate showed a good correspondence between the nominal and measured functional activity over the entire concentration range explored, while the other two products (Refacto AF and Kogenate) showed a progressive reduction of the measured activity compared with the nominal concentration. The intra-assay standard deviation of the measured activity of these products increased as a function of their concentration, especially for Kogenate and Refacto ( Fig. 5 ). Overall, the findings described above strongly suggest that the occurrence of aggregation processes, randomly developing as a function of the product concentration, could be responsible for the observed discrepancy between the measured and nominal FVIII activity.

### Activation of FVIII by Thrombin

**Fig. 6:**
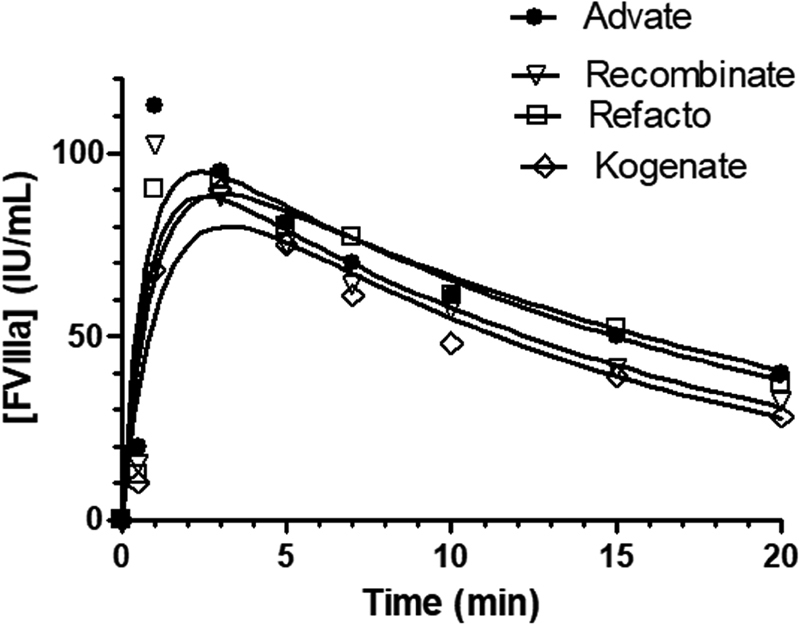
FVIIIa activation by thrombin of Advate, Recombinate, Kogenate, and Refacto AF. The *continuous lines* were drawn using the best-fit parameters [FVIII] _max_ , *k*_1_ , and *k*_2_ of Eq. 2 listed in Table 2 . In all cases, FVIII preparations were used at 100 IU/dL and thrombin at 20 nM. Experiments were performed in duplicate using different lots of the products (SD: 6 ± 2%), and average values are shown. SD, standard deviation.

The kinetic experiments aimed at investigating the ability of thrombin to activate the various FVIII preparations were analyzed by Eq. 2, whose best-fit parameters are listed in Table 2 and shown in Fig. 6 . Under the experimental conditions used in this study, the maximum FVIIIa generation peaked between 0.5 and 1 minute, with the rate of activation much higher than the inactivation rate ( Table 2 ). The activation rates showed moderate but significant interspecies differences with pseudo-first order activation rate in the order: Advate > Recombinate > Refacto = Kogenate. The inactivation rate constants were not significantly different among the different products, as shown in Table 1 .

**Table 2:** Best-fit kinetic rate constants derived from the application of Eq. 2 for FX activation by thrombin-activated FVIII from Advate, Recombinate, Kogenate, and Refacto AF

Parameter	Advate	Recombinate	Kogenate	Refacto AF
[FVIIIa] _max_ (IU/mL)	108 ± 17	103 ± 17	100 ± 15	103 ± 15
*k*_1_ (min ^−1^ )	1.41 ± 0.62	1.27 ± 0.6	0.85 ± 0.3	1.07 ± 0.4
*k*_2_ (min ^−1^ )	0.05 ± 0.02	0.06 ± 0.02	0.07 ± 0.02	0.049 ± 0.016

## Discussion

In the present study, we investigated some basic biochemical and biophysical properties of three commercially available rec-FVIII concentrates. Both SE-HPLC and DLS findings showed that these marketed rec-FVIII products, reconstituted following the procedures indicated by the producing companies, contain various amounts of aggregated FVIII molecules. These particles show significantly different hydrodynamic diameters, ranging from ∼100 to 1,000 nm. These solutes, too large to be attributed to monodisperse FVIII molecules, are removable with 220 nm cutoff filters and likely reflect the presence of unstable aggregates and/or partially unfolded forms. Whether the formation of these aggregates randomly depends on either the production and storage conditions of each lot or the method of reconstitution is unknown. In DLS experiments a significant inter-lot variability (10–15%) in the amounts of these large aggregates was observed. Notably, the presence of FVIII aggregates has been recently found using analytical ultracentrifugation in Advate, Helixate, and Kogenate. 16 Notably, in the present study, Advate was the only product in which it was possible to identify a more definite pattern of monodisperse forms of FVIII molecules ( Fig. 4 ). This behavior may derive from the specific conditions used to express the protein in CHO cells, in which Von Willebrand Factor (VWF) is co-expressed with the rec-FVIII to stabilize the latter. This strategy could favor the attainment of a correct conformation of FVIII, exploiting chaperon-like functions of VWF that would counteract unfolding and aggregation processes of FVIII molecules. A lower presence of protein aggregates in the Advate formulation was also found in sedimentation velocity analytical ultracentrifugation measures, as recently reported. 16

The above findings raised the question as to the presence of FVIII components with a very high hydrodynamic size could even affect the functional properties of the rec-FVIII concentrates. The activity of the various FVIII products was significantly altered as a function of the factor concentration. This finding agrees with the hypothesis that, being the formation of molecular macroaggregates a bimolecular process, it directly depends on the concentration of the reactants. Furthermore, the kinetics of protein aggregation may be an order of magnitude faster than folding kinetics, causing a significant fraction of the protein to be inactivated/unavailable. 17 Therefore, the process of protein aggregation may even favor a partial loss of their biochemical activity, as experimentally shown. According to the lower presence of aggregated particles, Advate (and its progenitor Recombinate) did not show a significant divergence of the FX activating capacity as a function of its nominal concentration. At variance with Advate, the other recombinant products Kogenate and Refacto partially lost their activity progressively as a function of their concentration, reaching a detrimental reduction of activity at ∼150 IU/dL nominal concentration. Moreover, the presence of molecular aggregates affected also the kinetics of FVIII activation by thrombin, as shown in Fig. 5 . In these experiments, Advate, which contained the lowest amount of aggregated particles, showed the best kinetic profile with the highest pseudo-first order rate constant of its activation by thrombin. The presence of aggregates or denatured protein in recombinant FVIII formulations has been also hypothesized to favor the production of anti-FVIII antibodies. 16 18 The presence of this irreversibly aggregated material could explain, besides other well-known risk factors, 17 19 20 21 the higher prevalence of inhibitor formation in patients treated with recombinant FVIII preparations, especially the second-generation products. 6 7 Furthermore, the immunogenicity of protein aggregates increases as a function of their molecular mass. 22 23 It was speculated that the molecular aggregates of FVIII might have a defective binding to VWF and be more easily detected by the antigen-presenting cells that induce an immunological response with the production of inhibitors. 9 24 As remarked above, Advate showed a relatively lower content of protein aggregates compared with Kogenate and Refacto and this fact might contribute to explain the lower incidence of inhibitor formation in patients treated with this product as compared with Kogenate and Refacto, as suggested in early and more recent observational studies. 6 7 25 26 In addition, the results of this study outline also the risk that the reconstitution of the recombinant products at a relatively high concentration may affect their pharmacodynamics/pharmacokinetics, limiting in part the availability of functional FVIII molecules. The introduction in the clinical practice of new recombinant FVIII products, also including the extended-half-life (EHL) formulations, currently represents a useful opportunity to investigate their physicochemical properties concerning the presence of molecular aggregates, in relation to the results that will be obtained on previously untreated patients (PUPs) in RCTs. Thus, it seems timely to investigate these properties of both EHL and modified preparations of rec-FVIII. It could also be very intriguing to compare the presence of molecular aggregates in plasma-derived FVIII preparations, although the presence of high molecular weight VWF multimers in these pharmaceutical formulations renders experimentally impossible any attempt to unequivocally verify this point.

In conclusion, the combined use of SE-HPLC, DLS, and enzymatic assays showed that aggregates with very high molecular size are present in therapeutic preparations of rec-FVIII and affect their functional properties. Thus, the possible formation of large-size aggregates in rec-FVIII products, particularly in the second-generation products, should not be ignored as a source of both altered pharmacodynamics/pharmacokinetics and as a possible trigger of an immune response in PUPs.
